# Management of Edentulous Microstomia Patient: A Case Report and Classification System

**DOI:** 10.1155/2022/2686983

**Published:** 2022-02-01

**Authors:** Maria S. Abbasi, Naseer Ahmed, Adil Bin Irfan, Samar Al-Saleh, Tariq Abduljabbar, Fahim Vohra

**Affiliations:** ^1^Department of Prosthodontics, Altamash Institute of Dental Medicine, Karachi 75500, Pakistan; ^2^PhD Scholar Prosthodontics Unit University Sains Malaysia, School of Dental Sciences, Health Campus, 16150 Kubang Kerian, Kelantan, Malaysia; ^3^Department of Prosthetic Dental Science, College of Dentistry, King Saud University, 60169, Riyadh 11545, Saudi Arabia

## Abstract

Microstomia is an abnormally reduced oral aperture. In the literature, it is not classified by any particular size criteria, rather defined by its effects on function and esthetics. Prosthodontic management of edentulous patients with microstomia is a challenging task. Use of conventional methods for recording an impression and fabricating prosthesis is not effective in such patients. To fabricate well-fitting prosthesis, accuracy of impression recording important anatomic landmarks is essential. Formation of an exacting custom tray and diagnostic cast is critical for final impression accuracy. Provision of a well-fitting prosthesis in microstomia patient will restore esthetics, comfort, and function with oral and systemic patient wellbeing. This paper presents a case report of managing an edentulous microstomia patient with sectional removable prosthesis. Furthermore, it proposes a novel classification system for microstomia patients according to severity of the condition.

## 1. Introduction

Microstomia is a congenital, developmental, and acquired condition, which presents with reduced oral aperture resulting in esthetics and functional impairment [1, 2]. It is usually associated with congenital syndromes and autoimmune diseases that cause connective tissue disorder [3]. It is majorly reported as a result of scarring following surgeries and trauma (accidental, thermal, chemical, and electric burns) around the perioral tissues, facial burn injuries, and head and neck irradiation [3]. In a study by Patil et al., 38.2% of the reported microstomia cases were due to postsurgical complications and 41.1% cases were due to systemic sclerosis [4]. Mostly microstomia is associated congenitally with Freeman-Sheldon, Treacher Collins, and Pierre Robin syndromes [5]. The occurrence of microstomia congenitally has also been reported with an unknown cause [6].

Microstomia patients may present with clinical problems including severe facial scarring, reduced width and mobility of lips, and loss of elasticity and altered anatomy of the oral tissues, particularly thickening of the labial and buccal tissues and contraction of the tissues around the mouth [4]. These features affect oral health-related quality of life, i.e., compromised chewing, speech difficulty, and appearance. Furthermore, a diminished intraoral access will affect oral hygiene maintenance and manual dexterity. Additionally, it makes the provision of dental prostheses quite challenging [5], especially among edentulous patients, in which oral function, esthetic, comfort, and self-esteem are at its lowest [6].

Therefore, treating such cases of edentulism and microstomia is pivotal not only from a functional standpoint but also from a psychological perspective. The goals of prosthodontic treatment are to restore masticatory function, improve esthetics, restore vertical dimension, establish lip support and competency, reduce drooling, and improve articulation [7]. The need for a classification based on diagnosis and overall patient management is lacking. The existing classification on microstomia which is the “index of oral access^1^” (IOA) does not address vertical mouth opening, difficulty in prosthesis fabrication, manual dexterity of the patient, and treatment options.

Proposed strategies for microstomia patients include use of sectional or flexible trays for primary impressions [8]. Sectional custom trays for maxillary and mandibular final impression, flexible dentures, and sectional complete removable dental prostheses using various cross pins, bolts, and attachments are proposed by clinical experts for hard and soft tissue replacement [9, 10],^11^. However, management strategies for edentulous microstomia patients are limited and challenging, with lack of defined standards. Therefore, in this report, the authors propose a classification system according to the severity of microstomia (Table 1), to guide practitioners in formulating diagnosis and treatment options according to the complexity of microstomia. We also present a case report of an edentulous microstomia patient managed successfully with a sectional complete denture.

## 2. Case Report

**Figure 1:**
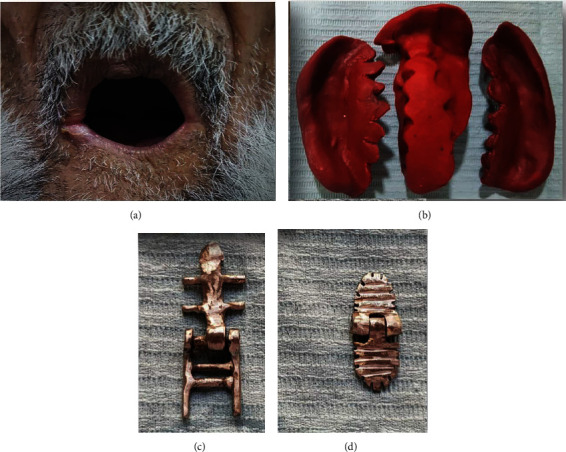
(a) Reduced oral access, 20 mm height and 32 mm intercommissural width. (b) Maxillary sectional primary impression. (c) Maxillary custom-made hinge. (d) Mandibular custom-made hinge.

A 62-year-old, male, edentulous patient reported to the prosthodontic department with functional inadequacy. On examination, he had reduced oral aperture, 20 mm interridge distance and 32 mm intercommissural width, along with inelastic buccal and labial tissues as a result of scarring and fibrosis following surgery around the corner of the mouth (Figure 1(a)). The patient had type 2 diabetes mellitus and was on oral hypoglycemic drugs. He displayed adequate manual dexterity and psychological status and belonged to class IV assessment of Prosthodontic Diagnostic Index [12] and DM-3 class (Table 1), based on the severity of microstomia. After consideration of all options, a plan to provide a sectional collapsible complete denture was suggested.

The primary impression for the maxilla was recorded in three sections. Firstly, the right and left ridge impressions were recorded separately with impression compound (Compound Red Cakes, Kerr Dental, CA, USA), trimmed 4-5 mm from the midline, and irregular notches were made at the mesial surface. Additional material was then added over the two halves to index the impression and complete the maxillary impression record (Figure 1(b)). The mandibular impression was recorded with impression compound in one piece. Sectional custom trays were fabricated from the preliminary cast with a stepped butt joint along the midline. Border molding was carried out with green stick impression compound (Compound Green Sticks, Kerr Dental, CA, USA) with right and left sectional trays in the maxilla and mandible. Following border molding, definitive impressions of both segments were recorded simultaneously with medium body polyvinylsiloxane (PVS) (Aquasil, Monophase, Dentsply Sirona, PA, USA) along with an index over the 2 segments for stabilization of the trays.

**Figure 2:**
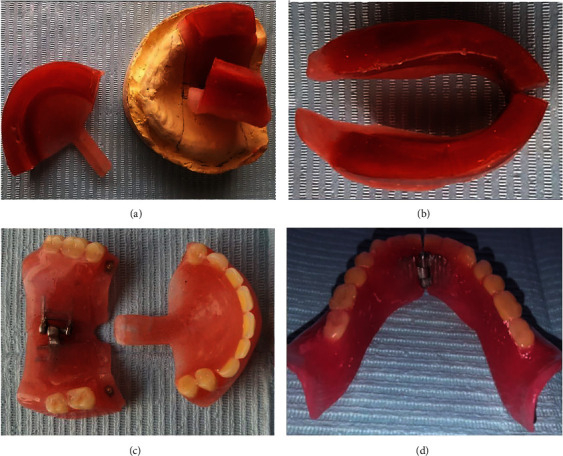
(a) Maxillary trial denture base and occlusion rim with custom-made hinge at midline of posterior segment. (b) Mandibular trial denture base and occlusion rim. (c) Sectioned maxillary prosthesis. (d) Sectioned mandibular prosthesis.

Maxillary final record base was fabricated in two segments (Nature-Cryl® HI-20ET-GC America, CA, USA), anterior and posterior; a custom-made Co-Cr hinge was fabricated following the design guidelines presented by Conroy and Reitzik [13] (Figure 1(c)). This hinge was incorporated in the midline as far back as possible in the posterior segment making it collapsible in the horizontal plane. For stability and bracing of the posterior segment, an anterior segment was placed over it. Similarly, a mandibular record base (Nature-Cryl® HI-20ET-GC America, CA, USA) incorporating a custom-made Co-Cr hinge (Heraenium EH, Kulzer, Zaragoza, Spain) anterolingually in the midline was fabricated in one piece making the base collapsible in the horizontal plane (Figure 1(d)). Maxillomandibular relationship in centric relation was recorded using sectional resin base with wax rims (Figures 2(a) and 2(b)) and was transferred to the articulator. Semianatomic artificial teeth were arranged in bilateral balanced occlusion. Two ball attachments (Clix-Ball attachment, Preat Corp., ON, Canada) were incorporated in the posterior segment of the maxillary denture base in order to retain the anterior segment to the posterior segment. A dental surveyor was used to obtain parallel paths of insertion on both sides (Figure 2(c)). Two ball abutment housings (female component) (Clix-Ball attachment, Preat Corp., ON, Canada) on the tissue surface of the anterior denture segment were incorporated with autopolymerized acrylic resin (GC Pattern Resin-LS, GC America, CA, USA) (Figures 2(c) and 2(d)). The resistance provided by the slopes of the residual ridges and the tongue pressure was used to stabilize the collapsible-hinged mandibular complete denture.

At the denture insertion, overextension of borders and sharp edges were removed by relieving the intaglio surface using disclosing paste (Pressure Indicating Paste, Keystone, Singen, Germany) and carbide acrylic bur (Patterson Dental, MN, USA) on a slow-speed motor. Occlusion was adjusted to achieve equilibration in static and functional position of the mandible. The patient was instructed and trained for denture assembly and removal. He was provided postinsertion instructions on hygiene, safe storage, maintenance, and regular follow-up to avoid mucosal ulcers and tissue inflammation, denture loss, and treatment failure.

## 3. Discussion

A planned and sequential approach is essential in treating microstomia cases. As the outcome is dependent on the clinical complexity of the case, along with the use of recommended materials and armamentarium, therefore, diagnosis and treatment planning are an essential component in the management [14]. A sectional complete denture prosthesis was provided based on the patient's treatment motivation, oral hygiene, extent of tissue loss, economic status, and available treatment duration. Sectional tray primary impression was recorded using impression compound with carved indexing in the center to assemble it extraorally. It allowed for staged process to overcome minimal access and maintained accuracy of the anatomic record [15]. The impression compound used for primary impression also acts as a flexible impression tray, in case the impression requires retake and improvements. The PVS medium body final impression was made for increased accuracy, dimensional stability, and elastic recovery [16].

The presented technique involved the fabrication of a sectional maxillary denture (anterior and posterior) and a combined incorporation of CoCr hinge in the center and ball abutments at the periphery of the posterior segment. This allowed for two-part insertion and enhanced stability due to the engagement of ball abutments and central hinge [17]. It is pertinent to mention that the success of prosthodontic treatment provided is multifactorial and improvement in mouth opening, soft and hard tissue health, and discontinuation of habits, good oral hygiene, and regular maintenance of prosthesis are critical for good treatment prognosis. As the management of microstomia is multifaceted, a holistic management plan is difficult to execute in the absence of a comprehensive classification system.

The present paper also presents a diagnosis and management (DM) classification system developed by considering the index of oral access (IOA) as baseline [1], as the latter does not address vertical mouth opening, difficulty in prosthesis fabrication, manual dexterity of the patient, and treatment options. This DM classification will assist in diagnosis, monitoring disease progression, interoperator communication, and record keeping in relation to microstomia. The patients could be treated with a variety of prosthetic options from a range of fixed and removable prosthesis keeping in mind adequate patient comfort and acceptance. Additionally, patients with microstomia suffering with chronic oral manifestation could be treated reliably, if this strategy is adopted on a large scale.

In spite of all, in the current era of digital development, it is believed that microstomia management will become more convenient and efficient. Intraoral scanning, computer-aided design and manufacturing (CAD-CAM), and 3D rapid prototyping can be utilized to produce precise sectional dentures. The hassle of recording manual impression, dental cast replication, and designing can be replaced with a digital workflow. Nevertheless, the use of dental implant-supported fixed prosthesis can add up to improve oral function and patient satisfaction.

## 4. Conclusions

The DM classification presented will assist in diagnosis and management of microstomia patients. The use of sectional removable dentures in the rehabilitation of edentulous patients with microstomia is effective; however, treatment prognosis is dependent on patient motivation and adaptation, case complexity, prosthodontic technique, technical skill, and maintenance.

## Figures and Tables

**Table 1:** Classification systems based on diagnosis and management for microstomia patients.

Class	IOA severity	Vertical mouth opening	Accessibility/visibility	Treatment options	Prosthetic fabrication difficulty	Manual dexterity
DM-1	Mild	Minimally compromised: 31-35 mm	(i) Denture-bearing areas of the mouth are fully accessible and visible (ii) Impressions and JRR can be recorded easily	(i) Conventional removable dentures (ii) Implant-supported prosthesis (iii) Flexible dentures	Not technique sensitive	Adequate
DM-2	Moderate	Moderately compromised: 21-30 mm	(i) Denture-bearing areas of the mouth have moderately compromised accessibility and visibility (ii) Moderately difficult to record impressions and JRR (modification of the tray/technique is required^∗^)	(i) Surgical correction (ii) Prosthodontic mx: (1) Implants supported/retained prosthesis (2) Flangeless prosthesis (3) Sectional complete removable dental prostheses—various cross pins, bolts, attachments, buttons, and Lego pieces can be used for the locking mechanism (4) Swing lock denture with cobalt-chromium framework	Moderately technique-sensitive design, moderately skilled lab/technician required	Fair
DM-3	Severe	Substantially compromised: 10-20 mm	(i) All the denture-bearing areas of the mouth have substantially compromised accessibility and visibility (ii) Extremely difficult to record impressions and JRR	(i) Surgical correction (ii) Prosthodontic mx: Sectional collapsible complete removable dental prosthesis	Highly technique-sensitive designs, highly skilled lab/technician required	Poor
DM-4	Extreme	Severely compromised: <10	(i) Denture-bearing areas hardly visible (ii) Impressions and JRR are not possible	Prosthetic rehabilitation not possible	—	—

DM = diagnosis and management; IOA = index of oral access; mx = management; JRR = jaw relation record; ^∗^flexible trays = sectional impression trays using die pins, sectional trays with interlocking-type handle or manually dispensing silicone putty.

## Data Availability

Data will be available on request to the corresponding author.
